# The Use of Plant-Derived Extracellular Vesicles in Regenerative Medicine Applied to Cutaneous Wound Healing

**DOI:** 10.3390/pharmaceutics17121531

**Published:** 2025-11-28

**Authors:** Victoria Pulido-Escribano, Marta Camacho-Cardenosa, Gabriel Dorado, José Manuel Quesada-Gómez, Alfonso Calañas-Continente, María Ángeles Gálvez-Moreno, Antonio Casado-Díaz

**Affiliations:** 1 Endocrinology and Nutrition Service-GC17, Maimonides Institute for Biomedical Research of Cordoba (IMIBIC), Reina Sofia University Hospital, 14004 Córdoba, Spain; victoria.pulido@imibic.org (V.P.-E.); marta.camacho@imibic.org (M.C.-C.); md1qugoj@uco.es (J.M.Q.-G.); alfonsoj.calanas.sspa@juntadeandalucia.es (A.C.-C.); 2 Departamento de Bioquímica y Biología Molecular, Campus Rabanales C6-1-E17, Campus de Excelencia Internacional Agroalimentario (ceiA3), Universidad de Córdoba, 14071 Córdoba, Spain; bb1dopeg@uco.es; 3 Centro de Investigación Biomédica en Red de Fragilidad y Envejecimiento Saludable (CIBERFES), 14004 Córdoba, Spain; 4 Department of Nursing, Pharmacology, and Physiotherapy, University of Córdoba, 14004 Córdoba, Spain

**Keywords:** extracellular vesicles, plants, wound healing, skin, hydrogels, regenerative medicine

## Abstract

The evaluation of the use of extracellular vesicles (derived from different cellular sources and mammalian fluids) in regenerative medicine has produced interesting results. This includes their great potential for the treatment of chronic skin ulcers, which is related to their effects on migration, proliferation, inflammation and angiogenesis, among other processes. However, large-scale production of mammalian extracellular vesicles may be limited by the need to maintain cell cultures continuously, without losing their ability to secrete extracellular vesicles with regenerative capacity. This may require complex and expensive infrastructures. It is therefore necessary to identify other possible, more efficient alternatives that can be easily transferred to clinical practice. Among these substitutes are plant-derived extracellular vesicles (PDEVs). Fortunately, they resemble those of mammals, playing a role in cell communications. As expected, their compositions depend on source tissues and the physiological conditions of the plants. They may carry numerous molecules with high biological activity. Interestingly, PDEVs are easy to obtain on a large scale, have good stability and are less immunogenic than mammalian-derived EVs. Numerous preclinical studies indicate that they can enhance chronic-wound healing through their immunomodulatory and angiogenic effects, among others. Thus, this review aims to describe the current state of knowledge on the potential therapeutic use of PDEVs in wound healing. It also describes the methods of obtaining and applying them, as well as regenerative processes in which they may intervene. The information provided shows the need to continue advancing knowledge about the production, isolation and mechanisms of action of PDEVs. This will allow new effective therapeutic strategies for the treatment of chronic cutaneous ulcers to be developed.

## 1. Introduction

### 1.1. Chronic Cutaneous Ulcers and Treatments

The skin, the body’s largest organ, is surprisingly multifaceted, with vital functions such as protection against external physical, chemical and biological aggressors. The skin is the first line of immune defense against infections, providing a shield against the harmful effects of solar ultraviolet radiation. Such energy from light is used to synthesize vitamin D3 and β-endorphins, which transmit painful and pleasurable stimuli, help regulate body temperature and maintain the body’s water and electrolyte balance [1].

Therefore, maintaining the function and structure of the skin is critical to health. As soon as a skin wound occurs, a sequence of events is set in motion to repair the wound: (i) hemostasis; (ii) inflammation; (iii) proliferation; and (iv) maturation/remodeling [2]. However, sometimes wounds do not progress according to the normal healing process. Instead, they may remain open in a chronic process, compromising the anatomical and functional integrity of the skin [3].

Chronic wounds represent a silent epidemic and a significant problem for public health systems worldwide [4]. Throughout their lives, between 1% and 2% of the population, especially older adults, will have suffered a chronic injury of mixed etiology [5,6]. As a result, these injuries place a significant burden on patients (affecting their quality of life and requiring caregivers), public health systems, healthcare providers and society as a whole [4]. Chronic ulcers increase the consumption of social assistance resources, including direct and indirect costs [6,7].

Due to the existence of different categories (pressure, neuropathic, venous and arterial), their prevalence is not well known. But a recent meta-analysis has concluded that mixed ulcers affect 2.21 people per 1000 inhabitants, and chronic leg ulcers affect 1.51 per 1000 inhabitants [8]. The prevalence of chronic wounds is expected to increase due to the aging population and the rise in diabetes, obesity and cardiovascular disease [9]. Regardless of gender, etiology of the wound, or age, patients with chronic wounds have higher morbidity [10] and mortality risk than the general population [11].

For all these reasons, promoting the healing process and shortening the duration of chronic wounds, especially those that are difficult to heal, are of utmost importance. Chronic wounds present a persistent inflammatory phase, preventing normal progression of healing. They are characterized by low levels of growth factors, unregulated protease activity and a high bacterial invasion. There is also an absence of progenitor cells and angiogenesis to supply nutrients and oxygen to the lesion [12]. The treatment of chronic wounds requires a thorough understanding of their etiology and physiology, as well as the healing process [13].

In clinical practice, a systemic framework is used for wound bed preparation, known as TIMER (an expansion of the original TIME framework), which stands for Tissue management, Infection and Inflammation control, Moisture balance, Edge of wound (Epithelial advancement) and Regeneration and Repair, which also involves social factors. This means that to prepare the tissue for healing, it is necessary to remove devitalized tissue, control inflammation and infection, maintain adequate moisture for healing and maintain healthy edges that promote epithelization and regeneration [14].

To promote wound healing, the first stage is debridement. It consists of removing host necrotic tissue, adherent dressing material, biofilm, slough, exudate and debris on the surface of the wound. There are several methods to accomplish this: surgical, mechanical, enzymatic, autolytic and even with maggot larvae [15,16]. During the healing process, excessive bacterial growth and infection need to be controlled. For that, topical antiseptic and systemic or local antibiotics are used [17].

Another important factor to promote wound healing is choosing an appropriate wound dressing. It should offer the following features: (i) protection from external microenvironments; (ii) exudate/moisture balance management, promoting epithelial migration and matrix deposition; (iii) pain reduction, reducing inflammation, evaporation and heat loss; (iv) esthetics, keeping the wound out of sight; and (v) compression, which is essential in venous ulcers, promoting venous return [18]. Today, some products are available for chronic ulcer treatment, such as hydrogels, hydrocolloids, paraffin gauzes, foams, semi-permeable films and silicone blends [19]. Sometimes, negative pressure therapy is used to promote granulation tissue formation, accelerate wound contraction, enhance local perfusion, clear toxic exudates, and likely reduce surface biofilm [20].

### 1.2. Cell Therapy and Cell-Free Therapy

**Figure 1:**
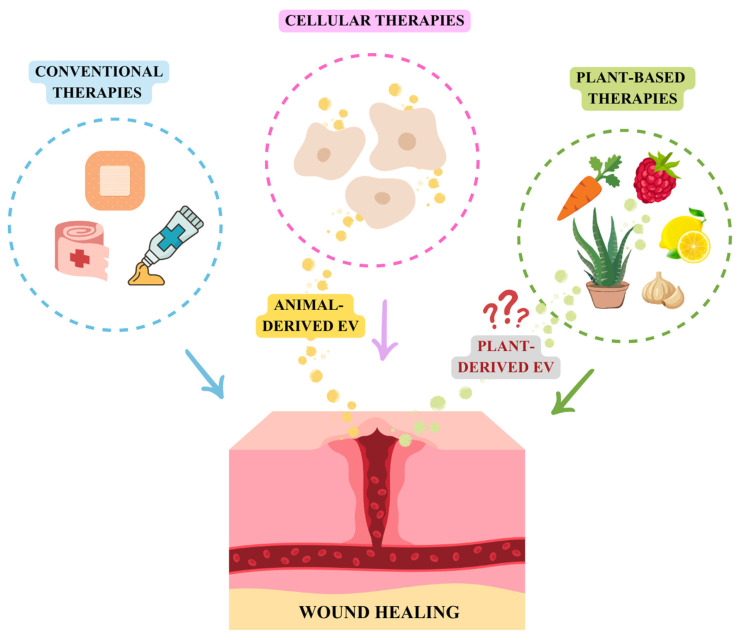
Different therapeutic strategies used in wound healing. In addition to conventional therapies, cell therapy and the use of plant extracts have yielded very promising clinical results.

Although conventional treatments like drugs and medical devices promote wound healing, some lesions remain refractory, leading to prolonged morbidity. Consequently, the demand for novel methods is increasing (Figure 1). In this sense, cell therapy emerges as a promising strategy. A variety of cells have been studied in this field to enhance wound healing: regulatory T-cells (T-regs), macrophages, platelets, fibroblasts and stem cells [18].

Fibroblasts play an important role in tissue homeostasis by reacting to signaling effectors and releasing growth factors and inflammatory cytokines, further exhibiting contractile abilities. Recently, some fibroblast-based cellular therapies have been clinically employed for wound treatment, as in Dermagraft [21], Apligraf [22] and TheraSkin [23].

In addition, stem cells have been extensively used in wound healing. An example is the commercialized Grafix, a cryopreserved placental membrane composed of fresh placental tissue, including extracellular matrix (ECM), growth factors and cells (epithelial, fibroblast and mesenchymal stem cells) [24]. Stem cells are characterized by their ability to self-renew and differentiate into various cell types [25]. Among them are embryonic stem cells, induced pluripotent stem cells (iPSC) and mesenchymal stem cells (MSCs), which are the most widely used in therapies.

Interestingly, MSCs have immunosuppressive effects and can be isolated from different sources, such as bone marrow, adipose tissue, the umbilical cord, the placenta, the endometrium, amniotic fluid, lung tissue and dermal tissue, among others [26]. They not only differentiate into functional cell types but also exert paracrine action by releasing cytokines and growth factors. This is known as secretome, which includes vascular-endothelial growth factor (VEGF), insulin-like growth factor 1 (IGF-1), hepatocyte growth factor (HGF), transforming growth factor β1 (TGF-β1), fibroblast growth factor 2 (FGF-2), prostaglandin E2 and bone morphogenetic protein-2 (BMP-2). They are involved in immunomodulatory, proangiogenic and antioxidant processes, aside from activating progenitor cells in injured tissues [27,28].

Cell administration could be systemic or by local injection. In the first case, cells may migrate toward injured tissues. However, in most cases, not all administered cells successfully reach target sites, as they can become trapped in other tissues. In the second case, when MSCs are delivered directly into the lesion, their arrival at the target site is ensured. In any of the scenarios, their survival may be limited, due to hostile microenvironments in damaged tissues, which may involve hypoxia, nutrient scarcity, etc. [29]. This is known as anoikis, which occurs when cells lose their adhesion to the extracellular matrix [30]. In addition, cell administration may present a series of problems. It may involve tumorigenic risks, infections, zoonotic transmissions, fibrogenic effects and embolic complications [31].

On the other hand, cell-free therapies represent a promising alternative, capable of overcoming such limitations. They are characterized by (i) low immunogenicity, (ii) convenient production, (iii) easy handling and (iv) long-term storage without loss of properties [31]. An example of cell-free therapy is using extracellular vesicles (EVs). These are heterogeneous particles, ranging in size from 20 to 1000 nm, enclosed by lipid membranes [32]. They are released by most cells and carry a diverse array of molecules, including peptides, proteins, lipids, nucleic acids and carbohydrates. These cargos may promote paracrine effects in recipient cells. They may involve inducing functional responses and promoting phenotypic and functional alterations under both physiological and pathological conditions [33]. Interestingly, preconditioning cells under different environments, such as hypoxia, can modulate both EV production and their molecular contents, as we have described [34]. Indeed, the potential clinical use of EVs in wound healing has been extensively evaluated in numerous studies, mainly preclinical ones, with promising results [32].

### 1.3. Plant Products to Promote Wound Healing

Another alternative, practiced since ancient times for healing purposes, is the application of medicinal plants (Figure 1). Products based on plant extracts are considered less toxic, causing fewer side effects compared to artificially synthesized chemicals, being safer for long-term wound dressing usage [35,36]. Their greater effectiveness lies in the bioactive phytochemical components that they may contain. Among these the following stand out: (i) phenolic compounds, which are organic chemicals with a hydroxyl group bonded to an aromatic ring (like flavonoids, tannins, anthraquinones and naphthoquinones); (ii) terpenoids (including saponins, which are a triterpenoid-glycoside subclass), alkaloids and essential oils. The latter are complex mixtures of volatile organic compounds. Interestingly, many essential oils contain phenolic compounds as a major component [37].

It should be also noted that chemical classifications may be “artificial”, with some kind of overlapping or ambiguity (as also happens with the classifications of biological entities). For instance, some compounds can share characteristics of both terpenoids and alkaloids. These compounds are associated with antioxidant capacity [38]; anti-inflammatory response [39]; and analgesic, antifungal, antibacterial [40] and anticancer properties [41].

Additionally, plant extracts like carvacrol may promote angiogenesis in human mesenchymal stem cells by influencing their differentiation, further enhancing their paracrine angiogenic activities [42]. In this sense, emodin, an anthraquinone found in various Chinese herbs like Chinese rhubarb (*Rheum palmatum*), Japanese knotweed (*Polygonum cuspidatum*) and tuber fleeceflower (*Reynoutria multiflora* syn. *Fallopia multiflora* and *Polygonum multiflorum*) induce collagen I synthesis in dermal fibroblasts [43]. Likewise, leaf extracts of mango (*Mangifera indica*) have anticoagulant and thrombolytic effects [44].

Olive-leaf extracts have also been investigated for their healing properties. In fact, our research group has evaluated their effectiveness and incorporated them into a hydrogel formulation (EHO-85). In vivo studies demonstrated an improvement in wound-healing properties via antioxidative activity, pH modulation and epithelialization, as compared with common hydrogels [45,46]. To conclude such investigations, a multicenter randomized clinical trial confirmed that this hydrogel promoted wound-area reduction, compared to a standard amorphous hydrogel, including in hard-to-heal ulcers [47,48].

Today, due to the benefits that plants offer, several laboratories are focusing on identifying their active compounds as potential alternatives for use in clinical practice. In this regard, recent research on plant-derived EVs (PDEVs) has attracted considerable attention. PDEVs are a heterogeneous group of vesicles with size ranges between 30 to 1000 nm, depending on source materials and isolation methods. They carry a variety of cargos, such as proteins, lipids, nucleic acids and other active molecules [49].

In contrast with mammalian EVs, PDEV lipid composition may include phosphatidic acid, phosphatidylcholine, digalactosyldiacylglycerol, monogalactosyldiacylglycerol and phytosterols. Phospholipids are involved in stability, vesicle liberation and intercellular communication, further participating in processes related to membrane fusions. Additionally, phosphatidylcholine and phosphatidylethanolamine play an important therapeutic role, including antioxidant, antiproliferative and anti-inflammatory effects [50]. PDEVs also contain proteins such as annexins [51], aquaporins [52] and heat-shock proteins like HSP60, HSP70, HSP80 and HSP90 [53]. They are molecular chaperones that assist in folding newly synthesized and misfolded proteins. That way, they help maintain overall cellular and protein homeostasis, including the structural integrity of plasma membranes, further controlling moisture permeability. In addition, they play an important role modulating plants’ stress responses.

The nucleic acids present in PDEVs are primarily microRNAs (miRNAs), which exhibit important cross-kingdom actions. Thus, they can interact with different biological entities like animals, plants, bacteria and viruses [54]. In fact, they have been observed to play an important role in regulating the expression of genes encoding inflammatory cytokines in human cancer-related genes [55]. In relation to the effect on wound healing, the molecules responsible for the therapeutic activity of PDEVs have not generally been identified in depth. However, in some plants, PDEV components involved in wound healing processes have been identified (Table 1).

In general, PDEV functions are involved in secretory processes to maintain cell growth, morphogenesis and intercellular communication, as well as defense against plant pathogens like viruses, bacteria and fungi [64]. Thus, they can transfer miRNA to fungi, blocking the expression of virulence genes. In mammals, PDEVs can modulate gene expression and thereby alter signaling pathways and physiological processes related to health or disease [65].

In comparison with animal EVs, PDEVs present some advantages, as described above. For example, the former are easier and cheaper to obtain, and they do not require extensive cell-culture steps for purification. They are also known for their resistance to enzymatic degradation, efficient absorption and low immunogenicity [63,66]. These properties have currently allowed PDEVs to be evaluated for their potential use in regenerative medicine. In this context, one possible clinical application of PDEVs is their use in the treatment of skin ulcers.

Thus, in recent years, studies on the effects of PDEVs in in vitro and in vivo wound-healing models have significantly multiplied. This shows that the scientific community currently considers PDEVs to have high potential for the treatment of skin ulcers and as alternatives to extracellular vesicles derived from mammals used in therapies. This is partly motivated by the better cost–benefit ratio that PDEVs may offer compared to mammalian EVs [67].

Taking these aspects into account, this review aims to present the information available on the potential of PDEVs for treatment of skin ulcers. In this review, studies with numerous plant species used to obtain PDEVs have been synthesized in relation to their roles in different phases of wound healing. The aim is to ascertain the versatility and multifunctionality of PDEVs for treatment of skin ulcers. In addition, this review highlights that encapsulation of PDEVs in hydrogels increases their therapeutic potential in wound treatment. Therefore, this review is aimed at serving as a tool to support and guide researchers in the development of new therapeutic strategies for treatment of skin ulcers.

To prepare this review, once the eligibility criteria had been defined—using the terms exosomes, extracellular vesicles or nanovesicles and plants and wound healing–two researchers independently conducted a rigorous literature search in the Web of Science database, the PubMed database and the Google Scholar search engine.

Once the search was complete and all relevant literature had been collected, articles were selected that described rigorous in vitro and/or in vivo studies evaluating the effect of PDEVs on different aspects related to the cells involved in wound healing or interventions in animal models of skin-wound healing. For the final selection, the researchers critically read and evaluated the quality of the manuscripts.

## 2. Biogenesis of PDEV

Due to the presence of cell walls in plants, the biogenesis of PDEVs differs from the one of animal EVs. According to their origins, they are classified as multivesicular bodies (MVBs), exocyst-positive organelles (EXPOs), vacuoles or autophagosomes. MVB are defined as late endosomes containing intraluminal vesicles, which are generated when membranes of the endosomes fold inward and bud into the lumen. Their formation is regulated by the endosomal sorting-complex required for transport (ESCRT). Intraluminal-vesicle cargos can either be degraded upon fusion with lysosomes or vacuoles or can be released as exosomes, if the multivesicular bodies blend with plasma membranes [68].

MVBs can deliver intraluminal vesicles to vacuoles, which can then fuse with plasma membranes to release their contents [69]. There is evidence that after bacterial infection, plants recruit vacuoles to fuse with plasma membranes, secreting vacuolar defense-molecules into extracellular spaces, to combat pathogens [70]. On the other hand, autophagosomes are double-membrane vesicles. They transport damaged or unneeded cellular components into vacuoles for recycling. To do that, they first fuse with MVBs to form amphisomes, which subsequently fuse with vacuoles [71].

EXPOs are morphologically distinct from MVBs, being also independent of endosomes and autophagosomes. The former are defined as double-membrane organelles, involved in unconventional exocytosis in plant cells. They form directly in cytoplasms, being identified by their Exo70E2 proteins. Their outer membranes fuse with plasma membranes, thus releasing their contents into extracellular spaces. Such vesicle diameters range between 200 and 500 nm. They allow the secretion of proteins without signal peptides, being involved in mechanisms of cellular defense and communication [72].

## 3. Methods for Isolating PDEVs

**Figure 2:**
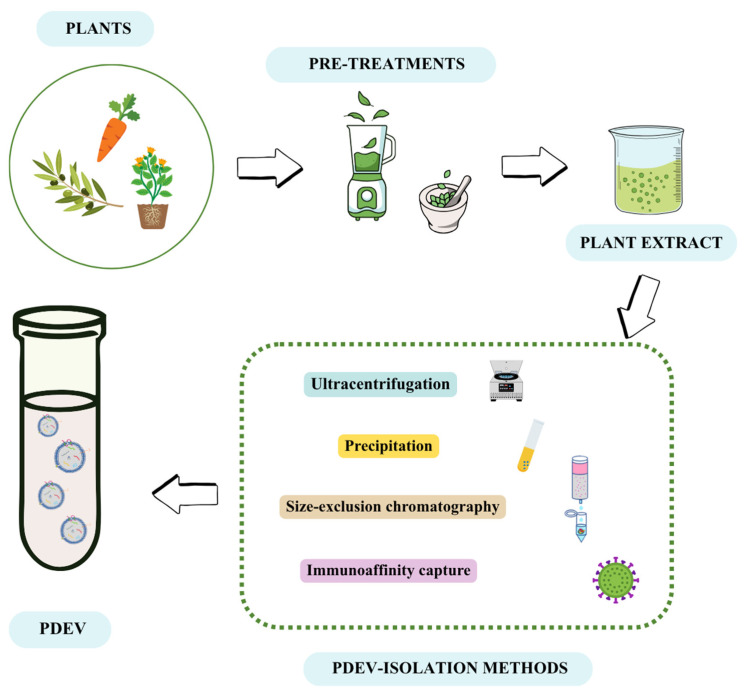
Methods for PDEV isolation. Any part of the plant (leaves, fruits, roots, etc.) is suitable for PDEV extraction. Extraction requires previous pretreatments, like crushing, liquefying, grinding or pressing. Next, ultracentrifugation, precipitation, size-exclusion chromatography or immunoaffinity capture, among others (including combinations of them), can be used for the isolation and purification of PDEVs.

Isolating PDEVs requires a suitable method depending on the purpose, whether experimental or clinical. PDEVs can be obtained from a wide variety of sources such as stems, leaves, roots and fruits [73,74,75]. Therefore, pretreatments may be needed. Specifically, fruits can be juiced or blended. Alternatively, leaves or stems can be disrupted in the presence of phosphate buffer saline (PBS) until a juice is obtained. In general, tissue grinding and pressing treatments could be useful in these cases [76]. Then, different methods for separating PDEVs can be carried out depending on their size, density, charge, solubility and surface-protein distribution. Among them, ultracentrifugation, size-exclusion chromatography, ultrafiltration, precipitation and immunoaffinity are the most used (Figure 2) [77].

### 3.1. Differential and Density-Gradient Ultracentrifugation

Ultracentrifugation (UC) is considered to involve centrifugal forces from 100,000× to 1,000,000× *g*. Ultracentrifugation separates by both size and density. The method used determines which property is the primary factor. Differential ultracentrifugation separates based primarily on size and density together, while density-gradient ultracentrifugation can be used to separate by density alone, or by a combination of size and density. Differential ultracentrifugation is one of the most widely used methods of segregating components of solutions. It is simple and does not require PDEV labeling, which helps prevent contamination. However, it requires long periods of time, expensive equipment and several centrifugation steps, which may affect final yields [78].

First, a low-centrifugation step (300× to 400× *g*) is necessary to remove the main part of larger/heavier debris. Then, higher centrifugations, between 3000× to 10,000× *g* for 20 to 40 min, are performed to further remove smaller extracellular and cellular debris, including aggregates and apoptotic bodies. The resulting supernatant is further ultracentrifugated using higher speeds (100,000× to 200,000× *g*) and a longer duration (1.5 to 2 h) to obtain PDEVs [79]. Yet, PDEV preparations obtained in such a way may contain contaminants. These include other vesicles, proteins and nucleic-acid aggregates. For that reason, subsequent purification steps may be necessary, such as microfiltration or using sucrose-density-gradient ultracentrifugation [80]. Currently, there are no standardized protocols for configuring centrifugal *g*-acceleration, rotor type, rotor sedimentation angle and solution viscosity [81].

On the other hand, density-gradient ultracentrifugation (DGU) uses a preconstructed density-gradient medium within a centrifuge tube, depending on the size, mass and density of the PDEVs. In plants, this method is frequently combined with ultracentrifugation-based methods to optimize separation effects and ensure integrity of PDEV membrane structures [82]. Gradient centrifugation is complex and requires considerable manual effort and time, frequently causing low recovery rates. Additionally, expensive equipment is required [83].

### 3.2. Ultrafiltration

Ultrafiltration is an effective method for purifying EV, especially for large sample volumes. The method involves the use of semi-permeable membranes with specific molecular-weight limits [membrane filter cutoff, or molecular-weight cutoff (MWCO)], generally from 10 to 100 KD. Pressure is applied to these membranes using centrifugal acceleration, for example. The separation of PDEVs by size, relative to other molecules and compounds, allows them to be quickly concentrated and purified. This method can be used to process large quantities of plant material. However, if the starting extract is very rich in vesicles and various proteins, the filter can quickly become blocked, affecting the purification process. In addition, other contaminating compounds, such as proteins or lipoproteins, may be retained along with PDEVs. For this reason, to achieve greater purification of the PDEVs, this method can be used in combination with other techniques, such as ultracentrifugation [84].

### 3.3. Size Exclusion Chromatography (SEC)

Size exclusion chromatography (SEC) is based on columns with porous beds implicated in separation, according to the size of the PDEVs, from other biopolymers and small molecules. SEC columns can be found with diverse stationary phases, depending on their structure and chemical composition [85]. The main advantages of SEC are its high effectiveness, user-friendliness and time savings, which help enhance EV production efficiency while preserving their integrity. However, it is more expensive than other methods and may have lower resolutions. Sometimes, it is combined with other techniques (such as ultrafiltration) to concentrate samples [77].

### 3.4. Precipitation

Polyethylene glycol (PEG) can be used to carry out this method. It involves combining and incubating the sample with PEG, followed by low-speed centrifugation. PEG decreases EV solubility, promoting their precipitation [86]. This technique includes several advantages, like easy implementation, large sample capacity and high yields. However, isolated PDEVs may show low purity because other contaminants may also coprecipitate, leading to false positives [77].

### 3.5. Immunoaffinity Captureisolo

This method uses beads coated with antibodies that can recognize specific protein marker(s) exposed on EV membranes [87]. It is suitable for high-specificity studies, but is not advisable for use in large-scale applications due to its high cost and low yield [77]. This methodology is widely used for purification of mammalian EVs. Yet, its use for the isolation of PDEVs is limited because currently no membrane markers have been identified to characterize these vesicles [67].

Table 2 shows a summary of some of the most important characteristics of each of the methods described, such as the amount of sample required, the quantity and purity of the EVs obtained, and the cost, among others. These characteristics should be taken into account when selecting the most appropriate method for isolating PDEVs. 

## 4. Therapeutic Potential of PDEV

**Figure 3:**
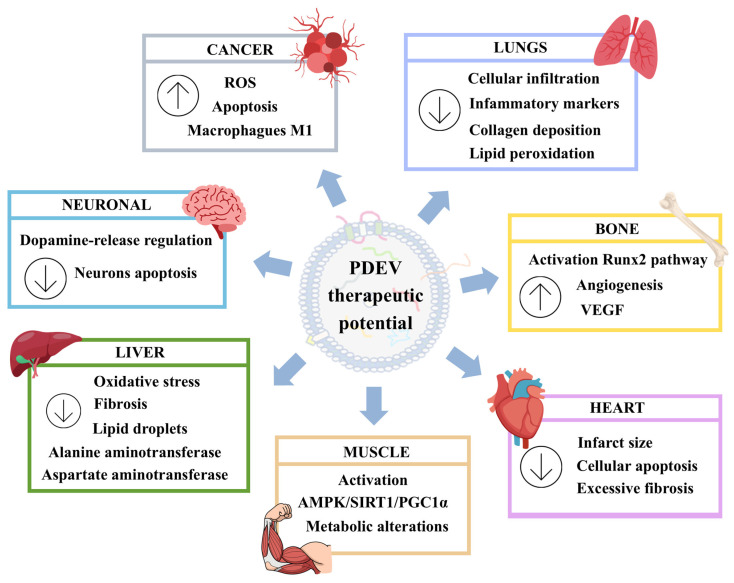
PDEV applications in biomedicine. PDEVs have been studied in diverse clinical applications, including cancer and regeneration of bone, nervous tissue, heart, lung, liver and muscle.

The bioactive compounds contained in PDEVs, which can affect numerous physiological processes in mammals, give them significant potential for treatment of various diseases. Furthermore, they can cross cellular barriers, such as the skin and blood–brain barrier. Additionally, they can be loaded with various molecules, including pharmaceutical drugs, for release in target tissues or organs. Therefore, they have generated considerable interest in the development of possible therapeutic applications [98]. Among these, their antitumor capacity stands out (Figure 3). In part, this is enhanced by the safety offered by the plant sources from which they are obtained, as well as the few side effects expected from their use [99].

An example has been carried out using balloon flower (*Platycodon grandiflorum*). EVs derived from this plant (PGEVs) have been used in in vitro and in vivo models of triple-negative breast cancer (TNBC). This disease shows high invasiveness, recurrence, metastasis and mortality rates. In vitro, PGEVs induced an increase in reactive-oxygen species (ROS) in tumor cells, which decreased tumor-cell proliferation and increased apoptosis. In addition, they promoted polarization of tumor-associated macrophages toward the M1 phenotype, inducing the release of pro-inflammatory cytokines. On the other hand, in vivo oral administration of PGEVs showed that they are stable in the gastrointestinal tract, accumulating in tumors and enhancing immune responses against tumor cells. This caused a decrease in tumor development, demonstrating the potential of PGEVs for treatment of TNBC [100].

Among the therapeutic properties of PDEVs are their low toxicity, biocompatibility and immunomodulatory effects. In addition, they can modulate cell proliferation and migration, as well as vessel formation [98]. All these processes are involved in tissue regeneration. Therefore, among the applications of PDEVs, their potential roles in regenerative medicine stand out. Specifically, they have been evaluated for treatment of musculoskeletal, neurodegenerative, cardiovascular, hepatic and respiratory diseases, among others (Figure 3). These include healing of skin ulcers, which is the main focus of this review, and will be described in detail in the following section.

In relation to bone regeneration, extracellular vesicles derived from yam (*Dioscorea* spp.) have the ability to promote the proliferation and differentiation of osteoblasts. That is accomplished through activation of the BMP-2/p-p38-dependent Runx2 pathway. Thus, oral administration in ovariectomized mice increased bone mineral density in tibias [101]. Recently, it was also shown that extracellular vesicles from “Horny Goat Weed” (*Epimedium brevicornu*) leaves administered orally to ovariectomized mice accumulated in bone tissue. They promoted bone formation through the induction of the VEGF signaling pathway and increased vessel formation [102].

Regarding the possible role of PDEVs in cartilage regeneration, it has recently been reported that EVs derived from tomato (*Solanum lycopersicum*) and grapefruit hybrid (*Citrus* × *paradisi*) juice promotes the differentiation of MSC derived from human adipose tissue into chondrocytes. Thus, the presence of these PDEVs in differentiation media enhanced expression of genes encoding chondrocyte markers such as *ACAN*, *SOX9* and *COMP* [103,104]. However, one study evaluating the effect of EVs derived from lemon hybrid (*Citrus* × *limon*) on chondrogenesis obtained the opposite results [103]. Such contradictory outcomes reveal the heterogeneity of the composition and biological activities of PDEVs, depending on plant origins. That should be taken into account in their possible therapeutic applications, including osteoarthritis [105].

Also related to the musculoskeletal system, the application of PDEVs may have effects on the maintenance and regeneration of muscle mass. Thus, EVs derived from berries of Chinese wolfberry (*Lycium barbarum*) were intramuscularly injected into the quadriceps of a mouse model of muscle atrophy. They increased the muscle cross-sectional area and grip strength. This effect was mediated by different mechanisms, including (i) activation of the AMPK/SIRT1/PGC1α signaling pathway; (ii) induction of changes in nucleotide and amino-acid metabolism; (iii) autophagy; and (iv) oxidative phosphorylation. All that contributed to muscle regeneration [106].

The antioxidant effects of PDEVs, as well as their ability to cross the blood–brain barrier, are particularly interesting. Such properties potentially allow EVs to be used as neuroprotective agents. In this way, they could be useful for the prevention and treatment of neurodegenerative diseases, which are characterized by increased oxidative stress [107]. Thus, proteomic analysis of the EVs of ferns (*Drynaria* spp.), such as the one known as “rhizoma drynariae” (*Drynaria roosii*), have been studied. Results suggest that they have potential in treatments of neurodegenerative diseases such as Alzheimer’s, Huntington’s and Parkinson’s [108]. In the case of Parkinson’s disease, EVs derived from gardenia (*Gardenia jasminoides*) have been shown to have high potential for use in its treatment. These EVs, both in vitro and in vivo, have a regulatory effect on dopamine release through inhibition of dopaminergic-neuron apoptosis [109].

The immunomodulatory, anti-inflammatory and antioxidant properties of PDEVs are useful for liver protection and regeneration [110]. For example, this was found with hemp (*Cannabis sativa*) sprouts. Hemp sprouts are of the same species as marijuana, but hemp is a specific cultivar grown for industrial and food purposes, distinguished by its low tetrahydrocannabinol (THC) content and genetic makeup. Thus, hemp sprout-derived exosome-like nanovesicles were orally administered to a mouse model of non-alcoholic fatty-liver disease. They reduced oxidative stress and fibrosis-marker proteins in the liver [111]. This anti-fibrotic effect in the liver has also been observed in animal models treated with EVs derived from tea (*Camellia sinensis)* leaves. These reduced the accumulation of lipid droplets in liver cells. Likewise, they reduced serum concentrations of both alanine aminotransferase and aspartate aminotransferase, which are markers of hepatotoxicity [112]. This demonstrates the potential of PDEVs for the prevention and treatment of liver diseases, such as non-alcoholic fatty-liver disease.

In respiratory diseases, the potential effect of treatment with PDEVs has also been evaluated. Thus, the anti-inflammatory effect of EVs derived from ginger (*Zingiber officinale*) has the ability to counteract the inflammatory process induced in severe acute respiratory syndrome coronavirus 2 (SARS-CoV-2) [113]. Also, in a mouse model with idiopathic pulmonary fibrosis (chronic and progressive disease with a poor prognosis), nasal administration of extracellular vesicles from such species produced a recovery in alveolar space size in the lungs. Additionally, it decreased cellular infiltrates, collagen depositions, expressions of genes encoding inflammatory markers, lipid peroxidations and apoptotic cells [114].

On the other hand, the regenerative potential of PDEVs in cardiac ischemia processes has also been studied in both in vitro and in vivo models. Thus, cardio myoblast cultures were subjected to oxidative stress induced with H_2_O_2_. It was found that the application of EVs from a carrot (*Daucus carota*) inhibited the increase in reactive-oxygen species and apoptosis [115]. Therefore, these results suggest that this type of EV could be used for the treatment of myocardial infarctions. In relation to in vivo models, fibrin gels were loaded with nanovesicles derived from *Lycium barbarum*. Their effects on cardiac regeneration in a mouse model of myocardial infarction were studied. Such gels improved both cardiac function and survival rates 14 days after myocardial infarction. In addition, such treatments reduced infarct size, cell apoptosis and excessive fibrosis, promoting cardiac repair. Furthermore, these effects were mediated by inhibition of the p38 mitogen-activated protein kinase (MAPK)/nuclear factor-kappa B (NF-κB) p65 (p38 MAPK/NF-κB p65) signaling pathway [116]. This shows how PDEVs can affect cell signaling pathways and how they regulate different physiological processes in mammals.

As described above, most studies conducted so far on the therapeutic potential of PDEVs correspond to preclinical studies. They were carried out in cellular and/or animal models. But the translation of the experimental results obtained to their medical practice in humans has already been carried out, or is in development. Thus, searching the ClinicalTrials database “<https://clinicaltrials.gov> (accessed on 9 October 2025)”, for ‘exosomes plant’ showed three clinical trials analyzing the effects of PDEVs. One of these is the “Study investigating the ability of plant exosomes to deliver curcumin to normal and colon cancer tissue” (ID: NCT01294072), currently in the recruitment phase. Curcumin is a product derived from turmeric (*Curcuma longa*). It acts as a chemopreventive agent in colon cancer, but its stability, solubility and bioavailability, when administered as a drug, are low. With this clinical trial, researchers want to ascertain if the use of PDEVs, as a vehicle for curcumin delivery, can increase the effectiveness of treatments.

Another clinical study is “Plant exosomes +/− curcumin to abrogate symptoms of inflammatory bowel disease” (ID: NCT04879810). Here, exosomes derived from ginger, as well as containing curcumin, are used. The aim is to determine if they can reduce the inflammatory processes associated with such disease. The third clinical trial is “Edible plant exosome ability to prevent oral mucositis associated with chemoradiation treatment of head and neck cancer” (ID NCT01668849). It evaluates the ability of exosomes from grapes (*Vitis vinifera*) to treat inflammation induced during radiotherapy and chemotherapy. Although these last two trials have been completed, there are still no scientific publications describing the results obtained. Therefore, despite these clinical trials, knowledge of the effects of PDEVs in human clinical practice remains limited. This represents a significant challenge for research on biomedical applications of PDEVs.

## 5. Application of PDEVs in Skin-Wound Healing

As described in the previous section, PDEVs have the ability to induce regenerative processes in mammals because they can promote progenitor-cell proliferation, cell migration and vessel formation and reduce inflammation, among other effects. These properties have led to the proposed use of PDEVs for the treatment of skin ulcers, as indicated below. This is because skin regeneration during healing is characterized by a series of coordinated and sequential phases in which PDEVs can act to promote such steps.

Maintaining healthy skin and regenerating it after injury is essential for the health of the body. It should be noted that the skin accounts for 16% of body weight, being the largest and arguably the most important organ in the body from a survival point of view. Its main function is to protect the body from external aggressions, such as ultraviolet radiation from sunlight and physical, chemical and biological agents. It also plays an important role in thermoregulation. Thus, vasoconstriction of blood vessels reduces heat loss (and shivering raises temperature), whereas sweat glands reduce temperature through sweating [117]. In terms of its structure, the skin is made up of three main layers: the epidermis, dermis and hypodermis. The epidermis is mainly composed of keratinocytes (95%). In the basal layer, they are proliferative (stem cells), while as they advance to the upper sublayers, they differentiate, progressively losing their nuclei, adopting an ovoid shape, and finally detaching [118].

The epidermis has invaginations housing sweat glands and pilosebaceous units, constituted by hair follicles associated with sebaceous glands. Pilosebaceous units play a role in the re-epithelialization of skin. Indeed, they contain epithelial stem cells, which give rise to basal keratinocytes. Below the epidermis is the dermis. This receives the main blood supply to the skin. Additionally, it contains most of the dermal appendages, such as the apocrine and eccrine glands, as well as hair follicles. Between the epidermis and dermis are interdigitations called dermal papillae. They increase the contact surface between the two layers, facilitating exchanges of nutrients and oxygen. The main cells in the dermis are fibroblasts. These are primarily responsible for the production of the extracellular matrix, which is predominantly composed of collagen [118].

When the skin suffers damage, the regeneration or healing process begins. Four overlapping phases can be distinguished in this process: (i) hemostasis; (ii) inflammation; (iii) proliferation; and (iv) maturation/remodeling [119]. Hemostasis begins at the moment the injury occurs, and its duration is a few minutes. In this phase, rapid vasoconstriction occurs and, through the action of platelets, a blood clot forms to prevent blood loss. The clot is rich in fibrin, which acts as a temporary matrix. In addition, during this phase, platelets release factors such as platelet-derived growth factor (PDGF), transforming growth factor beta (TGF-β), VEGF, tumor-necrosis factor alpha (TNF-α) and basic-fibroblast growth factor (bFGF), which play key roles in subsequent phases of healing.

The inflammatory phase begins during the first 24 h after the wound occurs. First, neutrophils infiltrate the injured area. These eliminate foreign bodies and pathogens, reducing the possibility of infection. They also release inflammatory mediators that attract and activate other cells involved in healing. These include monocytes and lymphocytes. Monocytes appear 48 h after the injury and differentiate into macrophages. In the wound, macrophages not only phagocytose debris and bacteria but also produce growth factors that promote tissue regeneration in following phases of healing.

Between 48 and 72 h, the proliferation phase, which can last up to 15 days, also begins. In this phase, damaged tissue is regenerated. The wound is closed through re-epithelialization, fibroplasia and angiogenesis. Fibroblasts contribute to wound closure by producing alpha smooth-muscle actin (α-SMA) and other extracellular matrix proteins, such as fibronectin and collagens. On the other hand, the process of angiogenesis is induced by local factors, hypoxia and decreased pH. It is essential for the formation of blood vessels that transport oxygen and nutrients necessary for tissue regeneration.

In the final remodeling phase, the extracellular matrix produced during previous phases is degraded, and type I collagen replaces the previous type III collagen. In addition, cell density and blood-vessel density decrease, resulting in tissue similar to uninjured skin. This phase, which begins 15 to 20 days after the injury, extends for months and can last up to a year [119].

As expected, disrupting the progress of these phases during healing negatively affects the regeneration of the skin and can lead to chronic ulcers. This usually occurs when the inflammatory phase is prolonged. In these cases, an excess of inflammatory cytokines promotes the degradation of growth factors and extracellular matrix proteins [120]. In addition, there is accumulation of reactive-oxygen species that increases oxidative stress and in turn promotes inflammation. That may create a vicious circle, preventing the proper healing of skin ulcers [121].

**Figure 4:**
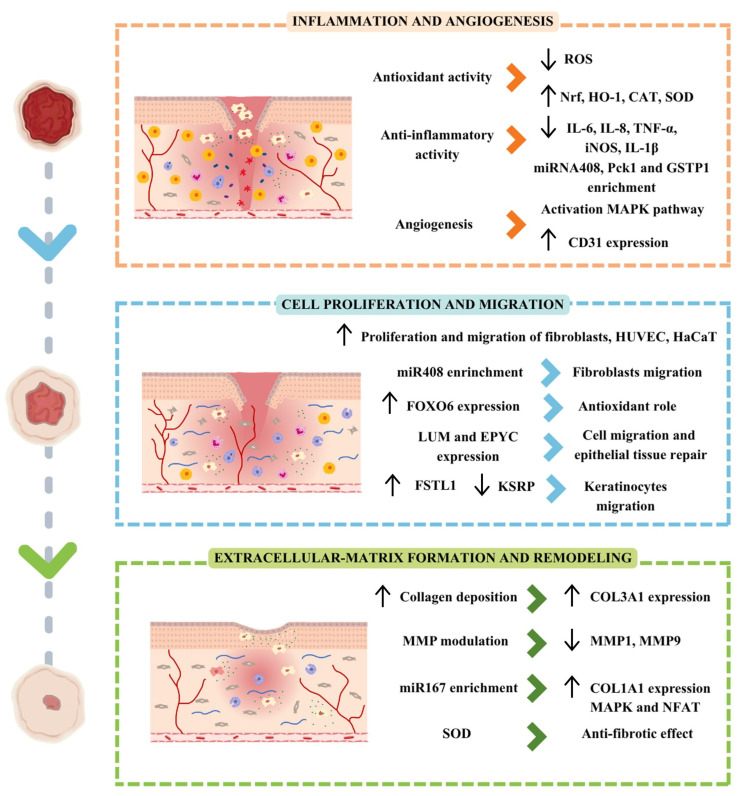
Effects of PDEVs on wound healing. PDEVs have the capacity to modulate the phases of wound healing, including inflammation, angiogenesis, oxidative stress, cell migration, matrix formation and remodeling. This is explained by their cargos, which may be involved in the induction/synthesis (↑) or repression/inhibition (↓) of different genes/proteins, respectively, that modulate important signaling pathways involved in various aspects of tissue regeneration, such as antioxidant defense, inflammation, proliferation, migration and cell differentiation.

PDEVs contain numerous bioactive compounds that can intervene in the modulation and induction of various processes related to skin healing. These include inflammation, proliferation, migration, angiogenesis and extracellular matrix formation, among others [122]. The combination of different compounds and metabolites, encapsulated in particles, allows PDEVs to affect different phases of the healing process. These properties make PDEVs potential therapeutic agents for promoting the healing of chronic ulcers. That can be accomplished through different mechanisms, with a single type of treatment. Thus, the following sections describe data available to date on how PDEVs from different sources can affect different phases and processes associated with skin healing (Figure 4 and Table 3).

### 5.1. Antimicrobial and Immunomodulatory Activity of PDEVs in Skin-Wound Healing

As mentioned above, prolongation of the inflammatory phase is one of the main causes of chronic ulcers. This may be associated with aging, as well as conditions such as diabetes and obesity [140]. Therefore, the antimicrobial, antioxidant and immunomodulatory effects of PDEVs are relevant in such scenarios. Such properties give these vesicles high potential for treatment of chronic ulcers.

The antimicrobial activity of PDEVs has been evaluated in EVs derived from different plants, such as Mongolian dandelion (*Taraxacum mongolicum*). These EVs have the ability to neutralize exotoxins produced by *Staphylococcus aureus*. This has been demonstrated in an in vitro hemolysis assay, where the disorder decreased from 80% to 4% in the presence of dandelion EVs [123].

During the inflammatory phase, cells such as neutrophils produce ROS, protecting damaged tissues from microbial infections and further stimulating other cell types for tissue regeneration. However, excessive ROS production can exceed the antioxidant defense capacity of cells. This causes oxidative stress and may lead to severe cell and tissue damage. This may promote chronic inflammation and impair healing [121]. On the other hand, plants possess important antioxidant systems. Unlike most animals, plants are mostly immobile and therefore have greater exposure to different environmental stressors, including extreme temperatures, ultraviolet radiation, salinity and pathogens. These factors may induce the formation of ROS. Therefore, plants have developed numerous systems to protect themselves from oxidative stress. These include production of phytochemicals such as vitamin C, phenolic compounds and carotenoids [141]. In this regard, PDEVs may be enriched in compounds of this type, as well as other factors with antioxidant capacity. Therefore, their applications in chronic ulcers may reduce oxidative stress and serve as an alternative treatment for this type of ulcer.

In vitro studies have also shown interesting results using HaCaT cells. HaCaT is a portmanteau combining the name of the lab where the cells were initially cultured (Hall) with the type of cells (CaT, for Caucasian keratinocytes). These human cells were spontaneously immortalized. Interestingly, different doses of PDEVs derived from several species, such as grapefruit, “Love Apple” (*Paris polyphylla* var. *yunnanensis*) and aloe (*Aloe vera* syn. *A. barbadensis*, *A. indica*, *A. perfoliata* var. *vera* and *A. vulgaris*), reduced intracellular ROS levels when cells were exposed to oxidative stress induced by H_2_O_2_ [57,124,125]. In the case of treatment with EVs derived from aloe, the antioxidant effect was mediated by the activation of the transcription factor nuclear factor erythroid 2–related factor 2 (Nrf2). This factor is the main regulator of genes encoding enzymes involved in oxidative stress defense, playing an important role in healing [142]. Aloe-derived EVs induced gene expression of *Nrf2*, heme oxygenase-1 (*HO-1*), catalase (*CAT*) and superoxide dismutase (*SOD*), in addition to SOD enzymatic activity. This indicates that these EVs contain factors capable of activating antioxidant defense mechanisms in keratinocytes [57].

The antioxidant effects of PDEVs—specifically, EVs derived from the fruit (pear) of a cactus (*Opuntia ficus-indica*), known as OFI-EVs—have also been evaluated in dermal fibroblasts. Among the suggested nutraceutical benefits of this fruit are its anti-ulcerogenic, neuroprotective, antioxidant, anti-carcinogenic, antiproliferative and hepatoprotective effects [143]. It is known that exposure of fibroblast cultures to H_2_O_2_ increases ROS levels. However, preincubation of cells with OFI-EVs for 24 h reduced ROS production about 1.4-fold. This effect was accompanied by a reduction in lipid peroxidation in cultures. It also increased both the mRNA levels of the *SOD2* gene and activity of its encoded SOD2 antioxidant enzyme. This indicates that treatment with OFI-EVs induces protection through the antioxidant system of dermal fibroblasts subjected to oxidative stress [62]. In addition to the antioxidant effect of OFI-EVs, these vesicles have anti-inflammatory properties. Thus, the human monocyte-leukemia cell line (THP-1) was pretreated with OFI-EVs for 24 h. Then it was stimulated with lipopolysaccharide (LPS) to induce an inflammatory response. Interestingly, the results showed a downregulation of the genes encoding interleukin-6 (*IL-6*), interleukin-8 (*IL-8*) and *TNF-α* pro-inflammatory cytokines, as well as a decrease in their encoded protein synthesis [62].

Additionally, in vitro inflammatory models were evaluated using the LPS-induced murine macrophage cell line (Raw264.7). Thus, such cells were exposed to EVs derived from “Love Apple”, soap aloe (*Aloe maculata* syn. *A. saponaria*) or *Aloe vera*. Such treatments significantly decreased the expression of pro-inflammatory cytokines, such as TNF-α, interleukin-1 beta (IL-1β) and IL-6 [59,125,126]. In the case of EVs derived from *A. vera*, it has been suggested that the anti-inflammatory effect is not mediated by the presence of phenolic compounds. This is because the study of the content of these compounds in nanovesicles showed very low levels compared to extracts obtained from this plant. In addition, the effect on the downregulation of the pro-inflammatory cytokine gene expression was greater with treatment with EVs derived from *A. vera* as compared to treatment with kaempferol and quercetin. These two polyphenols, which have anti-inflammatory activity, are also present in such plants. On the other hand, proteomic analyses revealed the presence of anti-inflammatory proteins, such as phosphoenolpyruvate carboxykinase (Pck1) and glutathione S-transferase P (GSTP1), in their EVs. Yet, the latter protein was not detected among proteins in plant extracts. That indicates a differential selection of molecules during EV biogenesis, which may influence their therapeutic properties [59].

In addition to carrying anti-inflammatory proteins, PDEVs can exert their immunomodulatory function through the transfer of miRNA. Interestingly, plant miRNA can bind to mammalian mRNA. That way, it can modulate different signaling pathways and biological processes, such as cell differentiation [144]. It has recently been shown that EVs derived from callus obtained using leaves of common camellia (*Camellia japonica*) contain high levels of miRNA408. In plants, they are mainly expressed in chloroplasts, where they regulate chlorophyll synthesis, among other processes [60]. Induction of inflammatory responses in HaCaT cells with TNF-α and interferon gamma (IFN-γ) was reduced in the presence of EVs from *C. japonica*. Interestingly, this inhibition of inflammation was mediated by the presence of miRNA408 in EVs. That was shown by the fact that treatments with miRNA408 also produced effects, which were similar to the ones of vesicles in HaCaT cells exposed to an inflammatory stimulus [60].

In the application of PDEVs in wound healing, it is pertinent to ask whether purity and the methods of isolation of nanovesicles could influence their properties, including those related to inflammation. In this regard, the isolation of EVs derived from lotus (*Nelumbo nucifera*) leaves by different protocols has been described. They included tangential-flow filtration (TFF), ultracentrifugation, density-gradient ultracentrifugation, size-exclusion chromatography and polymer precipitation (PP). Such methods generated different particle concentrations and purities, with TFF offering the best results for both parameters. Yet, no differences were found for the attenuation of inflammatory responses of RAW264.7 cells stimulated with LPS and treated with EVs obtained by different procedures. This indicates that the anti-inflammatory effect of lotus-derived EVs was not affected by the isolation method or the purity of the preparation. This finding is important if confirmed in EVs derived from other plants due to its implications in possible PDEV translational medicine, from basic science discoveries in the laboratory, to human clinical use [127].

The anti-microbial, antioxidant and anti-inflammatory effects of PDEVs in early stages of healing have also been evaluated using in vivo models. Thus, the anti-microbial effect of dandelion-derived EVs has been demonstrated in a mouse model in which *S. aureus* EVs were injected subcutaneously. This pathogen created massive blood exudation, ulceration and skin irritation in mice. Likewise, it increased the infiltration of inflammatory cells and production of pro-inflammatory cytokines such as TNF-α, IL-1β and IL-6. Yet, interestingly, pretreatment with dandelion EVs prevented all these symptoms. In such cases, the skins of treated animals exhibited a similar appearance to those of animals not exposed to *S. aureus*. This shows how dandelion EVs have the ability to neutralize the cytotoxic effects of such pathogens [123].

The antioxidant effects of PDEVs have been demonstrated, for example, with a preparation called OXY-ExoAloe. It consists of a mixture of EVs derived from neem (*Azadirachta indica*), *Aloe vera* and ginger. It is isolated from a combination of plant extracts in a ratio of 9:0.5:05, respectively. Interestingly, application of OXY-ExoAloe to wounds in rats (induced to be diabetic with streptozotocin) decreased lipid peroxidation and increased glutathione (GSH) levels. This, together with the decrease in *IL-6* gene expression in animals treated with OXY-ExoAloe, shows the antioxidant and anti-inflammatory effects of this PDEV preparation on wounds [128]. Additionally, EVs derived from mango ginger (*Curcuma amada*) have also shown anti-inflammatory effects when topically applied to skin wounds in a streptozotocin-induced diabetic mouse model. Treatment with these nanovesicles decreased gene expression of *IL-6* and the inducible isoform of nitric oxide synthase (*iNOS*). This may be associated with suppression of the inflammatory response of M1 macrophages [136].

In another in vivo wound-healing model in mice in which LPS was used to induce delayed healing, the effect of applying EVs derived from purple gromwell (*Lithospermum erythrorhizon*) was evaluated. In animals treated with EV, wound closure was 32% greater than in those treated with LPS alone. At the histological level, such treatment reduced the infiltration of inflammatory cells, which promoted healing processes. These animals showed greater collagen deposition and, therefore, more mature extracellular matrices than animals treated with LPS without EVs from such plant species [130].

As mentioned in the previous sections, numerous studies have evaluated the effects of plant extracts on wound healing. It is therefore interesting to compare the action of these extracts with those of EVs derived from the same plants. Recently, a study was conducted comparing the anti-inflammatory capacity of PDEVs with that of an ethanolic extract, both obtained from onion (*Allium cepa*). The study was carried out in a mouse wound-healing model. Both the PDEVs and ethanolic extract exhibited antioxidant effects on wounds, increasing SOD activity. However, the increase was mainly significant with PDEV treatment. Both treatments reduced the mRNA levels of nuclear factor kappa-light-chain-enhancer of activated B cells (NF-κB) p65 subunit (*NF-κB p65*) and nitric oxide synthetase 2 (*Nos2*), which are markers of inflammation and M1 macrophages. Additionally, such treatments increased levels of arginase 1 (Arg1), which is a marker of M2 macrophages. This indicates that both PDEVs and the extract have anti-inflammatory effects and promote the polarization of M1 to M2 macrophages. At the histological level, epithelialization was better in animals treated with the ethanolic extract. Therefore, this study highlights slight differences between PDEVs and the ethanolic extract in their effects on healing [58]. In this case, PDEVs had a higher antioxidant capacity than the ethanolic extract. This may be an advantage for the treatment of chronic ulcers. The previous study did not consider this, as it used a normal mouse model without pathologies associated with healing problems, such as diabetes or obesity.

### 5.2. Effects of PDEVs on Angiogenesis

The regeneration of tissue during healing requires the formation of new blood vessels. This is essential to supply the nutrients, oxygen and growth factors necessary to regenerate damaged tissues. Vessel formation is activated after tissues remain in hypoxic conditions, as a result of injury. Indeed, hypoxia activates the hypoxia-inducible factor-1 (HIF-1) transcription factor. This regulates the expression of hundreds of genes, including vascular-endothelial growth factor A (*VEGF-A*), directed at promoting angiogenesis and tissue regeneration [34]. However, angiogenesis is one of the processes that is reduced in chronic wounds, as it is difficult to heal. That is the case in diabetic foot wounds. Therefore, induction of vessel formation, mainly in this type of wound, is an important factor to consider in the development of treatments aimed at accelerating healing [145].

In this regard, it has been demonstrated that PDEVs from different plant species have the ability to increase the formation of tubular structures in endothelial cell cultures. Thus, treatment of human umbilical-vein endothelial cells (HUVEC) with EVs from grapefruit, cactus fruit, soap aloe or wheat (*Triticum aestivum*) in angiogenesis assays in Matrigel increased tube lengths, branching points and total loops as compared to untreated cells [62,124,126,131].

However, in most studies, the molecular basis of the angiogenic effects of PDEVs has not been described. Nevertheless, in some cases, the signaling pathways altered by the presence of PDEVs have been identified, which promotes vessel formation. Thus, EVs derived from Indian mulberry (*Gynochthodes officinalis* syn. *Morinda officinalis*) have been shown to increase proliferation, migration and tubular-structure formation in HUVEC cultures. Furthermore, in full-thickness skin-wound models in vivo, these EVs also have angiogenic effects. They increased gene expression of platelet endothelial-cell adhesion molecule-1 (*PECAM-1*), also known as cluster of differentiation 31 (*CD31*), which is an endothelial marker [66]. Proteomic studies and subsequent analyses of signaling pathways induced by treatment with Indian mulberry EV have been carried out. They identified the MAPK signaling pathway, being the most significantly enriched pathway. Thus, treatment with EVs activated protein kinases, including mitogen-activated protein kinase kinase 1 (MEK1, also known as MAP2K1) and mitogen-activated protein kinase kinase 2 (MEK2, also known as MAP2K2), which increased synthesis levels of yes-associated protein 1 (YAP1) and hypoxia-inducible factor-1-alpha (HIF-1α) [66]. Both proteins are involved in the activation of angiogenesis [146]. Consequently, their activation through treatment with EVs from Indian mulberry increased vessel formation, promoting healing in skin ulcers.

### 5.3. Role of PDEVs in Proliferation and Migration of Cells Involved in Skin Regeneration

The proliferative phase of skin healing is characterized by regeneration of new tissue in damaged areas of wounds. In this phase, cells responsible for regeneration must proliferate and migrate into damaged areas in order to perform their functions. Among the cells involved in this phase are fibroblasts, which are attracted to damaged areas by both factors produced by platelet degranulation and cytokines mainly secreted by M2 macrophages [147]. Fibroblasts in the wound are primarily responsible for synthesizing extracellular matrices. They are mainly made of collagen, elastin and proteoglycans. They replace provisional matrices, formed by fibrin in the previous phases. In fact, matrices synthesized at this stage of healing can be considered transitional in relation to the ones that will constitute the final dermis. The main component of such transitional matrices is a randomly organized network of type III collagen fibers. They act as a support for vessel formation and the epidermis. Subsequently, type III collagen is replaced by properly structured type I collagen fibers in the remodeling phase [119].

Endothelial cells are another type that proliferate and migrate into damaged tissues, involving formation of new blood vessels through angiogenesis. Hypoxia and pH changes, together with various factors produced by macrophages and fibroblasts, such as VEGF, bFGF and TGF-β, act as activators of endothelial function in this phase [120]. Re-epithelialization is another fundamental process during wound healing, in the proliferation phase. On the one hand, epidermal stem cells (located mainly in the bulge of hair follicles and in basal layers of interfollicular epidermis) migrate and proliferate, promoting wound closure. Additionally, keratinocytes at the edge of wounds decrease their adhesion to each other and to basal laminas in order to migrate over newly deposited matrices. In this way, these keratinocytes begin to migrate toward wound centers, while those located behind migratory tongues begin to proliferate. These processes are induced and regulated by various growth factors, cytokines, keratins, matrix metalloproteinases (MMP), chemokines and other macromolecules of extracellular matrices. Among the growth factors involved are epidermal growth factor (EGF), heparin-binding EGF-like growth factor (HB-EGF), transforming growth factor alpha (TGF-α) and keratinocyte growth factor (KGF) [148].

Proliferation and migration of cells, such as fibroblasts, endothelial cells and keratinocytes, largely depends on the presence of certain factors and on microenvironments in wound beds. In the case of chronic wounds, such an environment is unfavorable. Therefore, application of compounds or factors that improve such environment can help accelerate wound healing. In this regard, PDEVs derived from different species can perform this function, promoting healing, as reported by various studies. For example, in fibroblasts, treatment with EVs from Cape gooseberry (*Physalis peruviana*) or purple gromwell (*Lithospermum erythrorhizon*) in vitro increased proliferation and migration, as measured by 3-(4,5-dimethylthiazol-2-yl)-2,5-diphenyltetrazolium bromide (MTT) assays, Cell Counting Kit-8 (CCK-8) assays and scratch assays [130,132]. In the case of EV derived from soap aloe (a plant known for its content of phenolic compounds and flavonoids, with antioxidant and anti-inflammatory properties), concentrations of up to 5 × 10^9^ particles/mL have been shown to have no cytotoxic effects on HUVEC, RAW264.7 macrophages or dermal fibroblasts. Interestingly, in the latter, proliferation and migration by chemotaxis increased proportionally to the concentration of EVs in the medium [126].

Wheat EVs also promote proliferation and migration of human dermal fibroblasts, HUVEC and HaCaT cells in a dose-dependent manner, as do EVs from Indian mulberry on fibroblasts and endothelial cells. Concentrations of up to 200 μg/mL or 10^11^ particles/mL of these EVs, respectively, were not cytotoxic in such evaluated cell types [66,131]. However, EVs from other species, such as aloe, did not affect viability and proliferation of fibroblasts. Interestingly, they increased it by approximately 10% in HaCaT keratinocytes [59]. This shows how the effect of PDEVs depends on both the species of origin and the cell type treated. However, other in vitro studies with EVs from *Aloe vera* have shown that migration of HaCaT and dermal fibroblasts increased in the presence of these vesicles in a dose-dependent manner compared to untreated cultures, in scratch assays [57].

In general, studies that have demonstrated the positive effects of PDEVs on the migration of cells, such as fibroblasts and keratinocytes, have not usually explored in depth the molecular mechanism underlying these effects. In the case of EVs derived from callus from leaves of common camellia, it has recently been demonstrated that in vitro they promote migration of human fibroblasts. Additionally, it has been demonstrated that this is mediated in part by the presence of miR408 in such vesicles. In fact, this miRNA also significantly induced gene expression of both collagen type I alpha 1 (*COL1A1*) and collagen type I alpha 2 (*COL1A2*) in fibroblasts while reducing that of matrix metalloproteinase 1 (*MMP1*). These results suggest an important modulatory role of miR408 on fibroblast activity in the healing process [60]. However, human genes that can potentially interact with miR408, or those related to the effects observed in fibroblasts, have not been identified.

On the other hand, the potential of PDEVs to promote fibroblast migration in microenvironments of oxidative stress (such as the one in chronic ulcers) has been demonstrated. For instance, in the case of EVs derived from the fruits of cactus. Thus, human dermal fibroblasts reduced their migration capacity in scratch assays in the presence of H_2_O_2_. However, application of such EVs to cultures subjected to oxidative stress produced 85% wound closure in scratch assays after 24 h compared to 38% in untreated cultures. This suggests that the antioxidant effects of these EVs may play an important role in promoting granulation tissue formation in wound-healing processes under oxidative-stress conditions [62].

Also, in HaCaT keratinocyte cultures using in vitro migration assays, treatment with PDEVs derived from “Love Apple” increased wound closure by more than 40%. Additionally, they induced the expression of genes with antioxidant functions, such as forkhead box O6 (*FOXO6*). Likewise, they decreased expression of inflammatory interleukin genes, such as *IL-24*, *IL-1A* and *IL-1B*. In this case, transcriptomic analyses of HaCaT exposed to PDEVs showed interesting hits in Gene Ontology (GO) enrichment analyses: decrease in ‘negative regulation of cell population proliferation’ and increase in ‘positive regulation of cell migration’ and ‘extracellular matrix organization’. Additionally, upregulated genes included lumican (*LUM*) and epiphycan (*EPYC*). Both are related to cell migration and epithelial tissue repair. This highlights the ability of these PDEVs to promote such processes in skin healing [125].

In relation to protein synthesis, it was also observed that treatment of HaCaT keratinocytes with PDEVs had positive effects. Thus, concentrations of proteins related to cell migration increased. For example, EVs from grapefruit promoted migration of HaCaT cells in scratch assays. After 24 h of treatment with these PDEVs, cultures showed higher levels of (i) pro-migratory factors like “regulated-on-activation, normal T cell expressed and secreted” (RANTES); (ii) EGF; (iii) iIGF-1; and (iv) eosinophil-chemotactic protein (eotaxin). Likewise, they exhibited lower levels of anti-migratory tissue inhibitors of metalloproteinases-1 (TIMP-1) [124].

Another factor induced by application of PDEVs, being related to cell migration and wound healing, is follistatin-like 1 (FSTL1). This protein promoted keratinocyte migration in re-epithelialization processes during healing. Indeed, its inhibition led to chronic ulcers [149]. Interestingly, treatment of HaCaT cells in vitro, as well as wounds in animal-healing models in vivo, with EVs derived from mango ginger promoted keratinocyte migration and epithelialization mediated by induction of FSTL1. Curiously, this was accompanied by a decrease in the protein synthesis of KH-type splice regulatory protein (KSRP), which is an inhibitor of *FSTL1* gene expression. Thus, PDEVs from such species produced a pro-migratory effect on keratinocytes without exerting a significant effect on their proliferation [136].

The effects of PDEVs on cell migration have been compared to the ones produced by ethanolic extracts from the same plants. Both nanovesicles and extracts derived from tomato (*Solanum lycopersicum* var. *piccadilly*) accelerated wound closure in human keratinocyte, as well as in NIH-3T3-fibroblast monocultures, after six hours. However, the authors of this study highlighted that they used a protein concentration 16.6-fold higher in the extract, as compared to nanovesicles. They therefore concluded that PDEVs from such species exhibited a greater capacity than the extract to induce wound closure and cell migration [133].

As indicated in this section, there is considerable evidence suggesting that application of PDEVs to wounds is beneficial, improving cell proliferation and migration, and thereby promoting tissue regeneration. Nevertheless, some studies show that not all PDEVs have this effect. For example, EVs from mulberry had a positive effect on the proliferation of human microvascular-endothelial cells-1 (HMEC-1) but inhibited their migration [134]. Likewise, EVs from dandelion had no effect on fibroblast migration, but their inclusion in hydrogels had anti-microbial and anti-inflammatory effects when applied in vivo using a wound-healing model in mice, accelerating wound closure [123].

Recently, keratinocyte migration was evaluated using PDEVs derived from seven plants of the Zingiberaceae family and *Citrus* genus. In the case of PDEVs from Zingiberaceae plants (ginger and turmeric), all of them decreased viability. Yet, those from the *Citrus* genus, like grapefruit, lemon, sweet lime (*Citrus* × *limetta*), sweet orange hybrid (*Citrus* × *sinensis*), *Citrus* × *limon* and *Citrus* × *sinensis*, showed no cytotoxicity in HaCaT cultures at concentrations up to 500 μg/mL. Regarding the effect on HaCaT migration, EVs derived from ginger, turmeric, grapefruit and sweet lime exerted dose-dependent inhibitory effects. Those derived from lemon and sweet orange had no significant effects. Only EVs from ginger produced significant wound closure in scratch assays [136].

These studies show the heterogeneity of properties of EVs derived from different plants. Furthermore, they suggest that in order to identify EVs with greater clinical potential, it is important to evaluate the effects of EVs from different sources on different aspects and phases of the wound-healing process.

### 5.4. Effect of PDEVs on Extracellular Matrix Formation and Remodeling

Extracellular matrices are generated during the proliferation phase, mainly by the action of fibroblasts. These produce collagen, elastin, proteoglycans, and other components that make up the matrix. As mentioned above, type III collagen is the main component of the extracellular matrix. However, during the remodeling phase, it is replaced by type I collagen fibers, increasing the strength of the newly created tissue. This can take between 6 and 12 months [119]. As ECM production and maturation progresses, fibroblasts in the granulation tissue undergo phenotypic changes, differentiating into myofibroblasts. This is accompanied by remodeling of extracellular matrix proteins, which happens as a result of the action of proteolytic enzymes, such as metalloproteinases and serine proteases, produced by cells of the granulation tissue [150].

ECM synthesized during healing serves as a cellular support, fills spaces between cells, and provides the mechanical properties of the tissue. In addition to these functions, it also facilitates the microenvironment necessary for cellular processes, allowing tissue regeneration to occur. Thus, the composition of the ECM can influence cell migration, proliferation, survival, differentiation and morphogenesis [151]. Given the importance of adequate ECM production and maturation for proper regeneration of damaged tissue, alterations in the normal wound healing process can lead to pathological scarring. Thus, excessive collagen deposition can produce a hypertrophic or keloid scar, while insufficient collagen deposition can lead to an atrophic scar [119].

Among the effects that PDEVs can have on the healing process are those related to the formation and maturation of the ECM. They can affect both collagen deposition and the production of other proteins or enzymes involved in ECM synthesis or remodeling [132]. Collagen increases in scar tissue induced by PDEV treatments have been observed using in vivo models. For example, PEG-based hydrogels containing EV derived from ginger were applied to wounds in mice after streptozotocin-induced diabetes. This increased gene expression of type III collagen (*COL3A1*), as well as collagen accumulation in the ECM, as compared to wounds treated with hydrogels alone. This indicates an acceleration in wound healing when using EVs [136].

In addition to their effects on collagen synthesis, PDEVs can also modulate the production of MMP. These proteins degrade ECM components, including growth factors. Overexpression of their encoding genes is therefore associated with wounds with poor prognosis [152]. In fibroblasts, treatment with EVs derived from common camellia have been shown to decrease *MMP1* mRNA levels [60]. Meanwhile, in diabetic rats, application of OXY-ExoAloe inhibited synthesis of matrix metalloproteinase 9 (MMP-9) protein, which contributes to improved wound healing [128].

These effects may be mediated by the presence of certain miRNA in PDEVs. Thus, EVs derived from rock samphire (*Crithmum maritimum*) are enriched in miR167, among others. In plants, such miRNA is involved in the regulation of responses to stress, such as high salinity, water deficiency and diseases [153]. Curiously, in fibroblast cultures, miR167 induces/upregulates the gene expression of *COL1A1* and represses/downregulates that of *MMP1*. Indeed, this effect is similar to that obtained when cells are treated with EVs from rock samphire. This suggests that miR167 content in such vesicles is partly responsible for this result. Analyses of possible target genes in mammals, for miR167, have identified the gene encoding “protein phosphatase 3 regulatory subunit 2” (*PPP3R2*) as a possibility. The product of this gene is related to both MAPK and nuclear factor of activated T cells (NFAT), both involved in skin regeneration processes [61].

PDEVs may also be involved in the differentiation of fibroblasts into myofibroblasts. This process is important, because an excessive increase in myofibroblasts can lead to hypertrophic scars [154]. TGF-β1 is the main inducer of fibroblast differentiation into myofibroblasts [155]. The presence of TGF-β1 in fibroblast cultures activated the synthesis of alpha smooth-muscle actin (αSMA). That increased the contractile capacity in collagen matrices, which is characteristic of myofibroblasts. Interestingly, application of EVs from aloe in TGF-β1-treated cultures significantly decreased αSMA expression and collagen contractile capacity. This suggests that such EVs may decrease the development of undesirable fibrotic processes during wound healing of skin ulcers. Proteomic analyses of the composition of these EVs showed that the presence of SOD may be mediating the anti-fibrotic effects of these PDEVs [59,156].

## 6. Application of PDEV-Loaded Hydrogels in Wound Healing

PDEVs can be administered for therapeutic use in various ways, including orally, intravenously, subcutaneously or topically, among others [101,106,128,157,158]. In the case of wound treatment, topical application to damaged tissues is recommended in order to concentrate factors that promote healing in such areas. However, direct administration of PDEVs to the wound may reduce their efficiency due to rapid degradation, decreasing the time in which they can exert their effects. It is therefore advisable to use a carrier that prolongs their half-life, allowing for a longer sustained release over time.

Fortunately, hydrogels may meet such requirements. They are formed by polymers with a high capacity to absorb water, presenting characteristics similar to those of an extracellular matrix [159]. These properties of hydrogels offer many advantages for their application in wound healing. Such features include (i) biocompatibility and biodegradability; (ii) maintenance of a moist environment, reducing risks of scar formation; (iii) high capacity to effectively absorb wound exudates, which facilitates healing; (iv) high porosity, which permits oxygen transport and consumption, as well as better encapsulation of drugs, antioxidant agents, and particles such as EV; and (v) ability to facilitate sustained release of particles (including PDEV) into damaged tissues, promoting tissue regeneration and reducing the frequency of administration [160].

Recently, several studies have shown that encapsulation of PDEVs in hydrogels enhances their ability to promote healing in animal models. Among them, hydrogels based on oxidized alginate and chitosan, loaded with PDEVs derived from spearmint (*Mentha spicata*) as an antibacterial agent, have been described. In vitro studies showed that hydrogels loaded with these PDEVs, but not hydrogels alone, had antibacterial capacity against *Micrococcus luteus* and *Escherichia coli*. They were chosen because they are associated with infections in difficult-to-heal wounds. Such antibacterial effects were mediated by the ability of PDEVs to increase ROS in both bacteria [135]. Interestingly, in a rat model of a wound infected with *M. luteus* and *E. coli*, application of hydrogels loaded with PDEVs from spearmint accelerated healing. Thus, in the group of rats treated with the hydrogel only, yellow pus was observed after 3 days and, after 10 days, defects in the dermis with infiltration of inflammatory cells. In contrast, in the group treated with the hydrogel plus PDEVs, no pus was detected throughout the healing process, and after 10 days the skin was completely regenerated [135].

Other PDEVs with antimicrobial properties derived from dandelion have also been used in combination with a hydrogel consisting of gelatin methacryloyl (GelMA). This was studied in a wound healing model with *Staphylococcus aureus* infection in mice. The hydrogel exhibited good physical and mechanical properties, as well as controlled release of PDEVs into wounds. Experiments were carried out on infected wounds in which direct applications of PDEVs and hydrogel without particles were compared. Hydrogel loaded with PDEVs derived from Mongolian dandelion reduced inflammation, accelerated re-epithelialization and promoted the deposition and maturation of collagen fibers [123]. These studies showed the synergistic effects of PDEV-loaded hydrogels on healing and their potential for treatment of infected ulcers.

Hydrogel-based membranes containing PDEVs as bioactive compounds, such as dressings for wound treatments, have also been recently evaluated. For instance, chitosan-polyvinyl alcohol composite membranes with a mixture of PDEVs from three plant species, called OXY-ExoAloe (aloe, ginger and neem), exhibited flexibility, porosity and structural integrity, which promoted adequate oxygenation and moisture conditions in wound beds, thereby facilitating tissue regeneration. In streptozotocin-induced diabetic rats, application of OXY-NMAloe membranes significantly accelerated wound closure when compared to treatment with membranes without OXY-ExoAloe. This effect was the result of a reduction in oxidative stress and inflammation in wounds treated with OXY-NMAloe. Specifically, a decrease in lipid peroxidation, TNF-α and IL-6 transcripts, as well as an increase in glutathione were observed. In addition, such treatment also increased vessel formation and collagen deposition while inhibiting gene expression of matrix metalloproteinase 2 (*MMP-2*) and *MMP-9*. This reduced excessive degradation of extracellular matrices, facilitating regeneration of new tissue [129].

Similar results have also been obtained with hydrogels developed by incorporating polyvinylpyrrolidone and carboxymethyl chitosan through physical crosslinking. This hydrogel used encapsulated PDEVs from “Love Apple”. It was studied in vivo, with a mouse wound-healing model, demonstrating accelerated healing, decreased inflammatory-cell infiltration and increased collagen deposition when compared to wounds that were untreated, or treated with PDEVs or hydrogel alone. In this case, the release kinetics of PDEVs were studied. It was found that 61.3% had been released after six hours. This indicates a sustained release over time, allowing more efficient action in tissue regeneration than when applied directly to tissues. This study also evaluated the effect of such hydrogel on hemolysis. That was less than 2%, reflecting its biocompatibility for use in the treatment of skin ulcers [125].

The biocompatibility of a hydrogel with GelMA and dialdehyde starch containing PDEVs from lemon has also been evaluated in rats. This hydrogel did not produce apparent toxicity in organs such as the heart, liver, spleen, lung or kidney in treated animals [63]. In this case, interesting results were obtained studying the skins of diabetic rats after 14 days of healing treatment. Thus, the hydrogel containing PDEVs increased the expression of genes encoding M2-type macrophage marker Arg-1 and decreased that of M1-type macrophages iNOS. Therefore, such treatment induced polarization of macrophages toward M2 or regenerative macrophages. This is associated with the observation in these animals of decreased inflammation, as well as increased expression of genes encoding angiogenic markers (*CD31* and *VEGFA*) and collagen production [63].

Interestingly, the versatility of hydrogels allows incorporation of different bioactive compounds into their structures. Thus, dual-chamber sprayable multifunctional hydrogel was developed. It has the property of in situ gelation through rapid crosslinking of tannic acid, ferric ions and acrylamide. Additionally, flos sophorae-derived carbon quantum dots and cactus-derived EVs were incorporated. Flos sophorae is the dried flower bud of the Japanese pagoda tree (*Styphnolobium japonicum*). Flos sophorae carbon is produced by high-temperature carbonization and is used in traditional Chinese medicine as an anti-hemorrhagic agent [161].

Cactus has also been used in gel preparations for treatment of skin ulcers [162]. The application of this hydrogel in acute wound and burn models in mice has shown that it accelerates wound closure, promoting angiogenesis and hair-follicle regeneration. Additionally, it reduces inflammation, induces polarization of macrophages from M1 into M2 and decreases fibrosis. Furthermore, in vitro whole-blood coagulation assays were also carried out. Contact with the hydrogel containing the two bioactive components significantly accelerated coagulation. While coagulation occurred in 8 minutes in the control group, the hydrogel reduced it to 1.5 min. This value was also lower than that obtained with carbon quantum dots alone (4 minutes), or with hydrogel alone (2 minutes). Therefore, the incorporation of cactus PDEVs into such hydrogel, together with carbon quantum dots, positively affected wound healing. That was observed from the hemostasis phase to the regeneration of the extracellular matrix. Therefore, it has significant therapeutic potential in human clinical practice, for treatment of skin ulcers [137].

As demonstrated by the studies described above, incorporation of PDEVs into hydrogels may have synergistic effects on accelerating wound healing in animal models. Most studies described how oxidative stress or inflammation in wounds decreased, or how vascularization and extracellular matrix formation in regenerated tissue improved. However, few studies investigated in depth the signaling pathways affected by such treatments. In this regard, hydrogels using recombinant human Type III collagen were constructed, grafted with 3-inophenylboronic acid, to which PDEVs derived from chameleon plant (*Houttuynia cordata*) were incorporated. Wounds in diabetic mice treated with the hydrogel displayed anti-inflammatory, angiogenic and collagen-deposition effects, which were more pronounced than those in the wounds treated with just PDEVs or hydrogels alone. This was reflected in faster healing in the group treated with hydrogel plus PDEVs [138].

In that study, a transcriptomic analysis was also performed. Results showed that the interleukin (IL-17) and T helper 17 (Th17) signaling pathways, involved in regulation of chronic inflammatory responses, were modulated in the group treated with hydrogels plus PDEVs [138]. In fact, the study suggests that the treatment stabilized the nuclear factor of kappa light-polypeptide gene enhancer in B-cells inhibitor alpha (IκBα). This blocks the transcriptional activity of NF-κB and, consequently, reduces inflammatory responses [163]. The Hippo-YAP signaling axis was also identified as part of the action mechanism of the hydrogel with PDEVs. This pathway is involved in immunomodulation, cell proliferation and fibrotic processes [164]. Therefore, its inhibition can reduce fibrotic responses, as well as excessive cell proliferation, during wound healing [138].

Another mechanism of action by which PDEVs released from hydrogels can act on wound healing is related to the activation of the Nrf2 signaling pathway. This induces the cellular response to harmful changes in microenvironments, including increased antioxidant defenses in response to increased oxidative stress [165]. Thus, PDEVs derived from coriander (*Coriandrum sativum*) were encapsulated in sodium alginate hydrogel. They had positive effects on wound healing in a mouse model, partly through the activation of Nrf2, which was associated with a decrease in inflammation and accelerated healing in wounds treated with the hydrogel [139].

To the best of our knowledge, there are no clinical trials that have evaluated the use of hydrogels containing PDEVs for the treatment of skin ulcers in humans. However, existing data in the literature show the high potential of these hydrogels for use as dressings in wound treatments. As described above, incorporation of PDEVs into hydrogels allows for their gradual release. That enables their bioactive components to act during all phases of healing. Thus, the anti-inflammatory, antioxidant, angiogenic, migration-promoting and extracellular matrix formation properties of PDEVs may enhance the regenerative capacities of hydrogels, as evidenced by the studies described in this section.

## 7. Conclusions and Future Perspectives

The potential use of PDEVs for the treatment of chronic ulcers has been demonstrated, mainly in preclinical studies conducted in vitro and in animal models, but not in published clinical trials, although three have been registered in the ClinicalTrials database, as indicated above. However, the efficacy and long-term effects of applying a product containing stem-cell EVs from Damask rose hybrid (*Rosa* × *damascena*; RSCE) from ExoCoBio “<http://www.exocobio.com> (accessed on 19 October 2025)” has recently been described. This product has been applied for treatment of various dermatological conditions in humans, such as traumatic wounds, surgical scars and atrophic-acne scars. Although the study described only one case for each condition, results showed that the treatments produced benefits, and its use was proposed for wound healing and scar treatments [166]. Therefore, the preclinical nature of most research and the scarcity of clinical studies show that the development of possible therapeutic use of PDEVs in skin-wound healing is still in its early stages.

One of the main challenges for the clinical use of PDEVs in wound healing is to deepen our understanding of them—mainly about the pathways governing both their formation and interaction with target cells. This knowledge is essential for optimizing treatments and formulating compositions aimed at treating wounds according to their etiology. In addition, it could enable the development of drugs, as well as the engineering of PDEV compositions through biotechnology, to increase their efficacy. To this end, advanced multi-omic studies are needed to identify the molecular bases of their biological actions. However, one limitation to these types of studies is the heterogeneity of PDEVs from different plant sources. This complicates obtaining and standardizing general results or conclusions. Thus, although most PDEV evaluated for their potential use in wound healing have antioxidant and anti-inflammatory effects, mulberry PDEVs have been shown to induce the expression of inflammatory cytokines and ROS in macrophages [134]. This could negatively affect wound healing.

Interestingly, extracts obtained from mulberry have antioxidant and anti-inflammatory properties. This has been linked to their rich content of flavonoids, such as kaempferol and quercetin [167]. This suggests that PDEVs derived from mulberry, unlike extracts, are probably not enriched in these flavonoids. Therefore, as noted by the researchers who identified these opposing effects between extracts and PDEVs, omic studies (metabolomic, transcriptomic and proteomic) are needed to identify possible mechanisms by which mulberry PDEVs increase ROS production and inflammation [134]. In this regard, it should be noted that the main secondary metabolites produced by a plant are not always represented in PDEVs. For example, PDEVs derived from oranges are not enriched in vitamin C and naringin, which are abundant in orange juice [168].

Furthermore, the effect of PDEVs may differ depending on the cell type or tissue treated. In this regard, PDEVs derived from lemons generate ROS production in tumor cells and induce apoptosis [169]. However, as previously described, these PDEVs have positive effects on healing [63]. Therefore, this diversity of effects must be taken into account when evaluating the possible clinical applications of these extracellular vesicles. The composition of PDEVs determines their biological activity. Depending on the plant of origin, the tissue from which they come and environmental conditions, PDEVs may be more or less enriched in certain metabolites and other molecules [170]. This determines the mechanisms by which they can modulate different signaling pathways in mammalian cells. For example, the content of small RNA is part of the mechanism by which PDEVs derived from plants such as Japanese parsley (*Peucedanum japonicum*) display anti-inflammatory activity [171].

Thus, the heterogeneity in the origin, composition and properties of PDEVs is a limitation for both the standardization and scalability of their production for clinical use. Therefore, it would be necessary to develop standardized protocols for isolation, purification and characterization of PDEVs. That should allow for the reproducibility and safety of their therapeutic actions. In this regard, it is advisable to incorporate Good Manufacturing Practice (GMP) standards. Likewise, parameters to control the quality of PDEVs and products containing them should be established. The latter is a significant challenge because there are currently no general markers in PDEVs, as there are in mammalian EVs (e.g., CD63 and CD9). Therefore, it is also important to build an international consensus to establish minimum criteria for the development of clinical applications based on the use of PDEVs.

The development of standardized protocols for the production and characterization of PDEVs must be accompanied by their scalability to enable sufficient amounts for clinical application. First, it is essential to determine the source of the PDEVs. If they come from cultivated plants, the abundance and ease of obtaining the raw material will be greater than if they require cell cultures, seedlings or calluses produced in the laboratory. Although, in the latter case, the starting material may be more homogeneous, the cost would be higher due to the need for more complex installations.

Therefore, in terms of cost/benefit, it is essential to evaluate the type of production of the plant material from which PDEVs will be obtained. As for isolation techniques, ultracentrifugation, which is mainly used in research, is not very scalable for industrial-level production. This may be due to several factors: (i) the high energy consumption required to move large and heavy rotors; (ii) time-consuming operation that reduces productivity; (iii) contaminants that may be co-precipitated in the process; and (iv) the high shear forces to which PDEVs are subjected, compromising their integrity and functionality [172]. Among the alternatives to ultracentrifugation, a combination of TFF and SEC currently stands out as a procedure that allows large sample volumes to be processed for the isolation of extracellular vesicles, with adequate purity. In addition, this methodology is GMP-compliant [173].

Once PDEVs have been produced, another factor to consider for their clinical use is their storage, which must guarantee their stability and functionality until their therapeutic use. The most common methods for storing EVs are freezing, lyophilization (freeze-drying or cryodrying: vacuum sublimation after freezing) and spray drying. Freezing at −80 °C or −20 °C is the most widely accepted method, and may incorporate cryoprotectants to maintain the stability of the vesicles [172]. Finally, in spray drying, the solution is atomized and, with the application of hot air, converted into a dry powder [174].

The use of preservatives can help maintain the stability of PDEVs for longer, and at less extreme-low storage temperatures (typically ranging from –40 °C to –86 °C). For example, it has been shown that PDEVs derived from Korean dendropanax (*Dendropanax morbifera*) leaves can be stored for four weeks at 4 °C in a stable and functional form, in the presence of Saliguard TMO, a specifically designed alternative to parabens and formaldehyde preservatives, consisting of an extract of star anise (*Illicium verum*), caprylyl glycol, 1,2-hexanediol and butylene glycol [175].

The methods of application of PDEVs for treatment of ulcers are another important aspect to consider. Hydrogels are a suitable vehicle for topical application, and the results obtained so far are promising. Nevertheless, it is important to define which compositions may be more biocompatible and more suitable for each application. This is relevant to avoid unwanted interactions between hydrogels and PDEVs and will depend both on the nature of the hydrogel and the PDEVs’ plant of origin.

In this regard, it may also be interesting to investigate other possible routes of PDEV administration, such as oral intake. Thus, PDEVs from different plants have shown resistance to degradation as they pass through the digestive system, maintaining their biological capacity [111]. Investigating such approach could open up the possibility of creating new formulations and strategies. That could be useful for treatments of patients with chronic cutaneous ulcers. For example, in vivo studies in diabetes models have shown that oral treatments with PDEVs can improve glucose metabolism [176]. In the case of wounds, such as diabetic-foot ulcers, this could have a positive effect on healing. However, these alternatives must be accompanied by more in-depth studies on the safety of PDEVs. These studies must consider their possible use over prolonged periods of time, taking into account the slow healing process of difficult-to-heal ulcers.

Another challenge in the use of PDEVs is the regulatory one. Because their cargos contain bioactive molecules capable of modulating biological processes, PDEVs are likely to be considered pharmaceutical drugs. However, they have significant differences from drugs whose composition is perfectly defined. PDEVs are multimolecular complexes obtained through multi-step processes. They present challenges similar to those of other plant-based preparations. Thus, compared to standard pharmaceutical drugs with a defined composition, PDEVs may vary between batches. Therefore, they require more detailed characterization, which can be more complicated and costly. In this context, PDEVs could be classified as herbal-medicinal products [177]. As such, they would have to comply with the requirements of national and international regulatory agencies, such as the United States Food and Drug Administration (FDA) or the European Medicines Agency (EMA).

In conclusion, due to their biocompatibility, low toxicity and ability to release bioactive molecules, PDEVs have high potential as part of new treatments. They could be aimed at healing chronic cutaneous ulcers, as demonstrated by most of the preclinical studies performed to date. However, their incorporation into routine clinical use still requires overcoming significant challenges. These include a better understanding of their mode of action at the molecular level, standardization of isolation protocols and industrial-scale production. Likewise, the development of randomized controlled clinical trials with appropriate designs within a solid and internationally agreed regulatory framework is needed.

## Figures and Tables

**Table 1:** Molecular components identified in PDEVs that affect wound healing processes.

Molecular Component	Molecules	Plant Source	Effect on Wound Healing	Ref.
LIPIDS	—	Ginger (*Zingiber* *officinale*)	Anti-inflammatory. It inhibits the activation of nucleotide-binding domain and leucine-rich repeat containing family, pyrin domain containing 3 (NLRP3) inflammasome.	[56]
PROTEINS	SOD	Aloe (*Aloe vera*) Onion (*Allium cepa*)	Antioxidant defense.	[57,58]
	Phosphoenolpyruvate carboxy kinase (Pck1). Glutathione S-transferase P (GSTP1).	Aloe	Anti-inflammatory.	[59]
	Thioredoxin-dependent peroxiredoxin.	Aloe	Antioxidant defense.	[59]
NUCLEIC ACIDS	miR408	Common camelia (*Camellia* *japonica*)	Cellular migration activation *COL1A1* induction. Anti-inflammatory.	[60]
	miR167	Rock samphire (*Crithmum* *maritimum*)	Cellular migration activation. *COL1A1*, *COL1A2*, *VEGFA* and *TGFB1* induction. *MMP1* inhibition.	[61]
METABOLITES	Arbutin, apigenin, syringic acid, chlorogenic acid, kaempferol-3-O-pentose, quercetin-3-O-glucoside, sinapic acid, ferulic acid, rutin, quercetin-3-O-hexose deoxyhexose, kaempferol-3-O-glucose, caffeic acid and isorhamnetin-3-O rutinoside.	Cactus (*Opuntia* *ficus-indica*)	Angiogenesis activation. Fibroblast migration activation. Antioxidant defense. Anti-inflammatory.	[62]
	D-Ribo-phytosphingosine, citric acid, alpha-keto-gamma-(methylthio)butyric acid, diphenylphosphine oxide, dimethyl sulfoxide and vitamin C	Lemon hybrid (*Citrus* × *limon*)	Angiogenesis activation. Fibroblast migration activation. Anti-inflammatory. Collagen deposition.	[63]

**Table 2:** Main characteristics of PDEV isolation methods.

	Differential Ultracentrifugation	Gradient Ultracentrifugation	Ultrafiltration	Size-Exclusion Chromatography	Immunoaffinity	Polymer Precipitation
Procedure	Separation according to their sedimentation coefficient.	Separation according to size and density, relative to other components.	Filtration approach based on particle size, utilizing membrane filters and pressure.	The sample moves through a porous stationary phase. Smaller molecules, due to their lower hydrodynamic radius, pass through the pores more easily and elute earlier.	Relies on the selective interaction between exosomal surface markers and antibodies fixed to a solid support.	A hydrophilic polymer interacts with water molecules surrounding EVs, decreasing their solubility and forming a precipitate.
Sample	Large sample quantity is required because EVs are lost when the supernatant is removed after each centrifugation.	Lower volume used, depending on the column capacity.	Large sample quantity.	Small and large sample capacity.	Lower sample volumes.	Large sample capacity.
Time	12 h.	24 h.	2–4 h.	2–4 h.	1–2 days.	2 h.
Yield	Moderate.	Lower than differential ultracentrifugation.	Improvement over centrifugation methods.	High-yield isolation.	Low.	High.
Purity	It depends on the speed, time and rotor. There are variations between batches.	High.	High.	High.	High.	Low.
Cost	Expensive equipment, cost effective in the long term.	Expensive equipment.	Low equipment cost.	Cost effective.	Expensive, antibodies are required.	Cost effective.
Scalability	Low portability.	Low portability.	Good portability.	Good portability.	Low portability.	Low portability.
Disadvantages	Centrifugal force can break up EVs and contaminants can also be carried away.	Centrifugal force can break up EVs.	Risk of shear-induced damage and particle loss from membrane clogging.	Additional methods for EV enrichment are required.	Isolates only the EV subpopulations that show the targeted markers, leaving other types uncollected.	Impurities such as protein clumps, non-exosomal EV and polymer residues.
References	[88,89,90]	[90,91,92]	[90,92,93,94]	[90,92,93,95]	[90,96,97]	[86,90,94]

**Table 3:** Preclinical studies that have evaluated the therapeutic potential of PDEVs from different plant species to promote wound healing of skin ulcers.

Species	PDEV Isolation Method	PDEV Dosage	Study Type	Main Effects on Wound Healing	Ref.
Indian mulberry (*Gynochthodes officinalis* syn. *Morinda officinalis*)	Ultracentrifugation	10^7^–10^11^ part./mL	In vitro In vivo	↑ Proliferation and migration of fibroblasts and endothelial cells ↑ Angiogenesis ↑ Wound closure ↑ Collagen deposition	[66]
Mongolian dandelion (*Taraxacum mongolicum*)	Ultrafiltration	10^8^–6 × 10^9^ part./mL	In vitro In vivo	Antibacterial effects ↓ Inflammation ↑ Wound closure ↑ Collagen deposition	[123]
Aloe (*Aloe vera* syn. *A. barbadensis*)	*Ultracentrifugation and ultrafiltration*	1–10 × 10^8^ part./mL	In vitro	↓ ROS in keratinocytes ↑ Fibroblast and keratinocyte migration	[57]
Grapefruit hybrid (*Citrus* × *paradisi*)	Aqueous two-phase system (PEG/DEX)	0.5–4 × 10^9^ part./mL	In vitro	↓ ROS in keratinocytes ↑ Proliferation and migration in keratinocytes ↑ Angiogenesis	[124]
“Love Apple” (*Paris polyphylla* var. *Yunnanensis*)	Precipitation	5–20 μg/mL in vitro 10 mg/mL in vivo	In vitro In vivo	↓ ROS in keratinocytes ↑ Migration in keratinocytes ↓ Inflammatory response in macrophages ↑ Wound closure ↑ Collagen deposition	[125]
Cactus (*Opuntia ficus-indica*)	Ultracentrifugation	5–20 μg/mL	In vitro	↑ Angiogenesis ↑ Migration of fibroblasts ↑ Antioxidant defense ↓ Inflammatory response in monocytes	[62]
Soap aloe (*Aloe maculata* syn. *A. saponaria*)	Precipitation	0.1–5 × 10^9^ part./mL	In vitro	↓ Inflammatory response in macrophages ↑ Angiogenesis ↑ Proliferation and migration of fibroblasts	[126]
Aloe	Ultracentrifugation	100–500 EV/cell	In vitro	↓ Inflammatory response in macrophages ↓ αSMA expression in fibroblasts ↓ Ability of fibroblasts to contract collagen matrices	[59]
Common camelia (*Camellia japonica*)	Ultracentrifugation	10^8^ and 10^9^ part./mL	In vitro	↓ Inflammatory response in keratinocytes ↑ Fibroblast migration ↑ *COL1A1* gene expression in fibroblasts ↓ *MMP-1* gene expression in fibroblasts	[60]
Rock samphire (*Crithmum maritimum*)	Ultracentrifugation	2.6 × 10^8^ 2.6 × 10^9^ part./mL	In vitro	↑ *COL1A1* gene expression ↓ *MMP-1* gene expression ↑ Fibroblast migration	[61]
Lotus (*Nelumbo nucifera*)	Ultracentrifugation Ultrafiltration Precipitation SEC	0.5–10 × 10^10^ part./mL	In vitro	↓ Inflammatory response in macrophages ↑ Proliferation and migration of keratinocytes	[127]
Neem (*Azadirachta indica*) Aloe Ginger (*Zingiber officinale*)	Ultracentrifugation	200 μl	In vivo	Antimicrobial activity ↑ Antioxidant defense ↓ Inflammation ↓ *MMP-2* and *MMP-9* expression	[128,129]
Purple gromwell (*Lithospermum erythrorhizon*)	Ultrafiltration	0.1–10 × 10^9^ part./mL	In vitro In vivo	↓ Inflammation ↑ Wound closure ↑ Proliferation and migration of fibroblasts ↑ Collagen deposition	[130]
Onion (*Allium cepa*)	Centrifugation	100 μg	In vivo	↑ Antioxidant defense ↓ Inflammation ↑ Macrophage polarization from M1 into M2	[58]
Wheat (*Triticum aestivum*)	Precipitation	30–200 μg/mL	In vitro	↑ Proliferation and migration of fibroblasts, keratinocytes and endothelial cells ↑ Angiogenesis	[131]
Cape gooseberry (*Physalis peruviana*)	Precipitation	2.5–500 μg/mL	In vitro	↑ Proliferation and migration of fibroblasts ↑ *COL1A1* expression ↓ *MMP-1* expression	[132]
Tomato (*Solanum lycopersicum* var. *Piccadilly*)	Ultracentrifugation	30–200 μg/mL	In vitro	↑ Fibroblast and keratinocyte migration	[133]
Mulberry black and white (*Morus nigra* & *Morus alba*)	Density-gradient ultracentrifugation	1%	In vitro	↑ Endothelial-cell proliferation ↓ Endothelial-cell migration ↓ ROS in endothelial cells ↑ Inflammatory cytokines in macrophages ↑ ROS in macrophages	[134]
Spearmint (*Mentha spicata*)	Ultracentrifugation	5 × 10^4^ part./mL	In vivo	Antibacterial effects ↑ Wound closure	[135]
Mango ginger (*Curcuma amada*)	Precipitation	60 μg/mL	In vitro In vivo	↓ Inflammation ↑ Wound closure ↑ Migration of keratinocytes ↑ Collagen deposition	[136]
Lemon hybrid (*Citrus* × *limon*)	Ultracentrifugation	20 μg/mL	In vitro In vivo	↑ Hemostasis ↓ Inflammation ↑ Macrophage polarization from M1 into M2 ↑ Wound closure ↑ Angiogenesis ↑ Proliferation and migration of fibroblasts ↑ Collagen deposition	[63]
*Cactus sp.*	Ultracentrifugation	100 μg/mL	In vitro In vivo	↑ Hemostasis ↑ Antioxidant defense ↓ Inflammation ↑ Macrophage polarization from M1 into M2 ↑ Wound closure ↑ Angiogenesis ↓ Fibrosis	[137]
Chameleon plant (*Houttuynia cordata*)	Ultracentrifugation	10^10^ part./mL	In vitro In vivo	↑ Proliferation and migration of fibroblasts ↓ Inflammation ↑ Wound closure ↑ Angiogenesis ↑ Collagen deposition	[138]
Coriander (*Coriandrum sativum*)	Density-gradient ultracentrifugation	10–40 μg/mL in vitro 10 mg/mL in vivo	In vitro In vivo	↑ Migration in keratinocytes ↓ ROS in keratinocytes ↑ Macrophage polarization from M1 into M2 ↑ Antioxidant defense ↓ Inflammation ↑ Wound closure ↑ Collagen deposition	[139]

## Data Availability

No new data were created or analyzed in this study. Data sharing is not applicable to this article.
